# Pediatric Type 1 Diabetes: Mechanisms and Impact of Technologies on Comorbidities and Life Expectancy

**DOI:** 10.3390/ijms241511980

**Published:** 2023-07-26

**Authors:** Flavia Urbano, Ilaria Farella, Giacomina Brunetti, Maria Felicia Faienza

**Affiliations:** 1 Giovanni XXIII Pediatric Hospital, 70126 Bari, Italy; flaviaurbano84@gmail.com; 2 Clinica Medica “A. Murri”, University of Bari “Aldo Moro”, 70124 Bari, Italy; ilafarella@yahoo.com; 3 Department of Biosciences, Biotechnologies, and Environment, University of Bari “Aldo Moro”, 70125 Bari, Italy; 4 Department of Precision and Regenerative Medicine and Ionian Area, University of Bari “Aldo Moro”, 70124 Bari, Italy; mariafelicia.faienza@uniba.it

**Keywords:** type 1 diabetes, children, continuous glucose monitor, subcutaneous insulin infusion, automated insulin delivery, cardiovascular disease, nephropathy, retinopathy, neuropathy, bone health

## Abstract

Type 1 diabetes (T1D) is one of the most common chronic diseases in childhood, with a progressively increasing incidence. T1D management requires lifelong insulin treatment and ongoing health care support. The main goal of treatment is to maintain blood glucose levels as close to the physiological range as possible, particularly to avoid blood glucose fluctuations, which have been linked to morbidity and mortality in patients with T1D. Indeed, the guidelines of the International Society for Pediatric and Adolescent Diabetes (ISPAD) recommend a glycated hemoglobin (HbA1c) level < 53 mmol/mol (<7.0%) for young people with T1D to avoid comorbidities. Moreover, diabetic disease strongly influences the quality of life of young patients who must undergo continuous monitoring of glycemic values and the administration of subcutaneous insulin. In recent decades, the development of automated insulin delivery (AID) systems improved the metabolic control and the quality of life of T1D patients. Continuous subcutaneous insulin infusion (CSII) combined with continuous glucose monitoring (CGM) devices connected to smartphones represent a good therapeutic option, especially in young children. In this literature review, we revised the mechanisms of the currently available technologies for T1D in pediatric age and explored their effect on short- and long-term diabetes-related comorbidities, quality of life, and life expectation.

## 1. Introduction

T1D is one of the most common chronic diseases in childhood, whose incidence is progressively increasing; it requires life-long insulin treatment and continuous health care support. A major goal of therapy is to maintain blood glucose levels as close as possible to the physiological range to avoid long-term adverse effects of chronic hyperglycemia [1]. In particular, blood glucose fluctuations have been recently identified as a risk factor for morbidity and mortality in patients with T1D [2]. The impact of glycemic variability on cognitive function and brain development has been demonstrated especially in individuals with early-onset diabetes [3]. The guidelines of the International Society for Pediatric and Adolescent Diabetes (ISPAD) recommended a glycated hemoglobin (HbA1c) level < 53 mmol/mol (<7.0%) for young people with T1D to avoid long-term microvascular and macrovascular complications [4].

**Figure 1:**
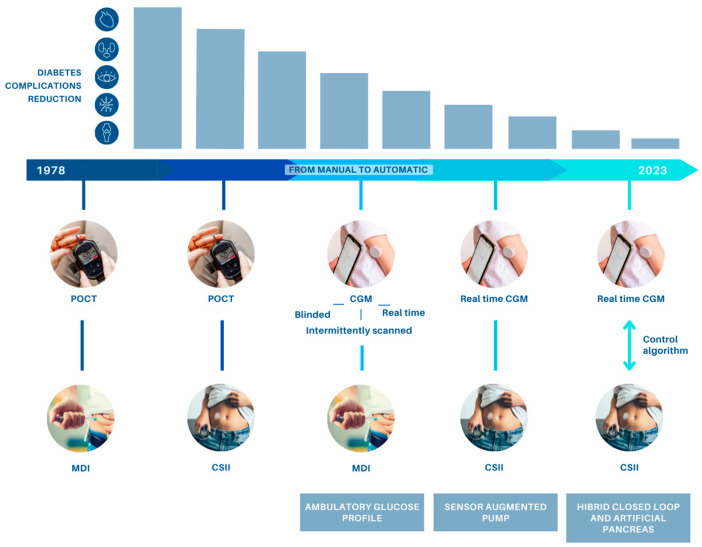
Device evolution for diabetes treatment; CGM, continuous glucose monitoring; CSII, continuous subcutaneous insulin infusion; MDI, multiple daily injections; POCT, blood glucose point-of-care testing.

In recent decades, numerous advances have highlighted the mechanisms of the disease, as technological advances have led to the development of automated insulin delivery (AID) systems, aiming to improve the metabolic control and the quality of life of patients with T1D. Continuous subcutaneous insulin infusion (CSII) combined with continuous glucose monitoring (CGM) devices connected to smartphones represent the therapy of choice, especially in young children [5]. In particular, CGM has proven to be increasingly reliable and effective in terms of improving HbA1c, reducing hypoglycemia, and increasing time in the target glycemic range. Furthermore, the artificial pancreas (AP) or closed-loop system (CLS) represents a future possibility in the pediatric population (Figure 1).

**Figure 2:**
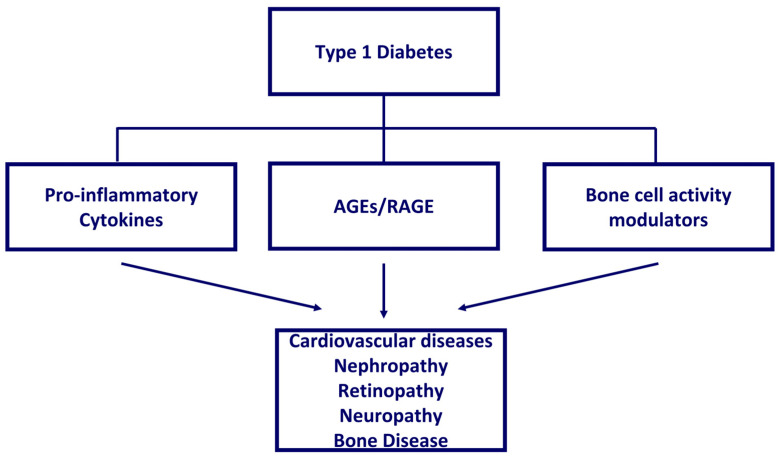
Main mechanisms associated with T1D pathophysiology and related complications.

In this literature review, we revised the mechanisms associated with T1D pathophysiology (Figure 2), reported the currently available technologies for T1D in pediatric age, and explored their effect on short- and long-term related comorbidities, quality of life, and life expectations.

## 2. T1D Pathophysiology

T1D results from the autoimmune destruction of the β-cells of the pancreatic islets [6] (Different mechanisms have been proposed to explain the pathogenesis. The “classical” theory looks at β-cells as passive targets of self-reactive CD4+ and CD8+ T cells. This idea supports the use of immune-target therapy for T1D prevention and management [7,8].

Consistently, in a clinical trial with teplizumab, targeting T cells, T1D patients at high risk of developing the pathology showed a three-year delay in progression with respect to the placebo group [8]. Additionally, using a monoclonal antibody against Tumor Necrosis Factor (TNF)-α (golimumab), a beneficial effect has been reported [9]. In detail, the improvement in β-cell activity, together with a reduced insulin requirement, has been demonstrated with respect to the placebo group [9].

More recently, a direct dysfunction of β-cells has been proposed for T1D development, with alterations of genes in these cells, including the *INS* gene itself [10]. Among the causes of β-cell dysfunction is the endoplasmic reticulum (ER) stress that culminates in β-cell apoptosis [11]. This is the cause of the activation of Unfolded Protein Response (UPR), a complex that can both maintain cell homeostasis (adaptive UPR) or activate cell apoptosis (terminal UPR) [12,13,14]. However, other mechanisms can concur in β-cell apoptosis, such as the perforin-granzyme pathway [15]. Additionally, necrosis has been investigated as an additional mechanism of T1D pathogenesis with unclear results [11].

Senescence recently attracted the attention of different researchers, although the triggers of this process remain unknown in T1D. Interestingly, it has been proposed that apoptosis and senescence are both involved in T1D pathogenesis [16]. Damage-induced senescent β-cells displayed signs of DNA damage response (DDR) involving phosphorylated histone H2A.X [17]. In β-cell growth block due to senescence is mediated by the increased levels of cyclin-dependent kinase inhibitors such as p21, p19, and p16 [18]. However, in T1D, the persistent DDR, together with the expression of these inhibitors, represents a unique profile leading to a distinct phenotype compared with aged β-cell or T2D [19,20,21,22]. Only a subset develops senescence-associated secretory phenotype (SASP) markers [18]. Interestingly, senescent β-cells are long-lived and continue to accumulate during disease progression [23,24]. Although senescence has a key role in T1D, to move with success versus a senescence-target therapy, some issues should be addressed.

β-cells are characterized by other dysfunctional states as proinsulin processing defects as well as a transdifferentiate status [25,26,27,28]. Mature insulin arises from the cleavage by different enzymes (PC1 and PC3), and interestingly, different studies reported a proinsulin processing defect in T1D, as supported by the augmented proinsulin/insulin ratio in islets, as well as persistent proinsulin secretion measured in patients’ sera [25,26]. Different studies demonstrated the altered expression of PC1/3 at both mRNA and protein levels. Other studies demonstrated that *INS* transcription is disrupted in T1D [27,28,29]. A bi-hormonal state has been demonstrated for β-cells, thus simultaneously producing glucagon and insulin, thus sustaining the trans-differentiation theory. This represents a particular subset of islet cells that curiously lacked typical α-cell markers [25,26].

Other mechanisms of β-cell dysfunction include defective autophagy and mitochondrial function [30,31,32,33,34]. However, due to the recent related discoveries, further studies are needed to realize clinical trials.

## 3. Continuous Glucose Monitoring Systems (CGM)

The test strips for POCT date back to 1978 [35]. The introduction of CGM systems changed the management of T1D by replacing the single-point measurements of capillary blood glucose concentrations and, thus, becoming the standard of care for children, adolescents, and young adults affected with diabetes [36]. CGM devices measure interstitial fluid glucose concentrations through subcutaneous glucose sensors at 1–15 min intervals using enzyme-coated electrodes or fluorescence technology. The accuracy of the new devices has significantly improved compared to the past, and differences between actual blood glucose levels and CGMs that occur in the hypoglycemic range and when glucose levels change rapidly are due to the approximately 5–10 min physiological delay between the flow of glucose from the intravascular to the interstitial compartment [37].

Accuracy is also affected by the time it takes for the sensor to react to glucose and by the use of digital filters to constantly transmit the sensor signal while converting the measured sensor signal into a glucose value [38]. Sensor performance may also be affected by biomechanical factors such as motion and pressure [39].

The parameter most frequently used for the description of the analytical performance of CGM systems is represented by the “mean (or median) absolute relative difference” (MARD) [40]. MARD is expressed as a percentage and represents the average of the absolute error between the CGM values and matched reference values. A small MARD number indicates that the CGM readings are close to the reference glucose value, whereas a larger MARD number indicates greater discrepancies between the CGM and reference glucose values. Typically, a CGM system with a MARD < 10% is considered to have good analytical performance [41,42,43]. In the home-use setting, the CGM system may produce higher average MARDs than during clinical studies [44].

Real-world studies demonstrated that CGM improves glycemic control and decreases hypoglycemia risk and the incidence of diabetes-related occurrences and hospitalizations [45]. Recently, the FDA approved a new “integrated CGM” (iCGM), which can automatically suspend or increase insulin infusion in response to current and/or predicted hypoglycemic and hyperglycemic events. As reported by the FDA, iCGM systems “are designed to reliably and securely transmit glucose measurement data to digitally connected devices, including automated insulin dosing systems” [45].

CGM systems can be classified into three categories: (1) blinded or professional CGM; (2) real-time CGM (rtCGM); (3) intermittently scanned CGM (isCGM) or flash glucose monitoring (FGM).

Blinded CGM is the first CGM device (Medtronic MiniMed CGMGold system) released by Medtronic in 1999. Professional CGM systems collect short-term glucose data which are not visible to the patient and provide health care professionals with data showing glucose patterns and trends. In addition to clinical practice, professional CGM systems are also employed in research settings to collect retrospective glucose data and to reduce potential bias.

Real-time CGM (rtCGM) systems automatically display glucose values at regular intervals and can use alarms when sensor glucose levels reach preset hypo- or hyperglycemia thresholds, as well as rate-of-change alarms for rapid glycemic variations. rtCGM systems transmit glucose data directly to smartphones. These data can then be stored on a web server (“cloud”) and used for remote monitoring by caregivers and healthcare professionals. In addition to traditional, self-implanted transdermal sensors with a lifetime of 6 to 14 days, a long-term implantable sensor for up to 6 months of use has been available with regulatory approval in the European Union since 2016. The Eversense CGM is currently approved only for use in subjects > 18 years of age. Unlike traditional CGM sensors, where glucose is measured using the enzyme-based electrochemical method, the Eversense implantable sensor uses non-enzymatic optical fluorescence. The next-generation Eversense CGM has a 180-day long-term wear time with daily calibration [46].

Intermittent Scanning CGM (isCGM) or Flash Glucose Monitoring System (FGM), FreeStyle Libre (FSL), does not automatically display glucose values at regular intervals but reports glucose levels when the user scans the sensor on a separate reader or Smartphone enabled for the communication protocol, near or above the sensor. Current interstitial glucose levels and glucose trend arrows are provided upon request, as well as a graph of current and stored glucose readings [47].

As with rtCGM, glucose data from isCGM can be transferred from a smartphone to a webserver for remote glucose monitoring by caregivers or health care professionals; the sensor can provide glucose values up to 14 days after a one-hour sensor warm-up period [46]. The second generation of FreeStyle Libre (FSL2) was approved in Europe in 2018 and in the USA in 2020. FSL2 sensors have higher accuracy measured by a MARD 9.2% and 9.7% for adults and children, respectively, and optional alarms when the glucose level is out of the target range [41]. To see the actual level, the user must scan the sensor. The third generation, the FSL3, is actually a rtCGM providing real-time alarms and real-time readings without the need to scan. It received a CE marking in 2020. The FGM system is easy to use, accurate, does not require any calibration during the 14-day lifespan, and is relatively inexpensive. Some disadvantages are the lack of alarms during hypoglycemia or hyperglycemia, although the new generation of the FGM will also provide alarms, as well as a lack of interaction with insulin pumps [47].

The growing spread of continuous glucose monitoring (both CGM and FGM) led to the introduction of measurements derived from sensor data as time in range (TIR), that is, the time spent in the glucose range 70–180 mg/dL, time above range (TAR) (>180 mg/dL) and time below range (TBR) (<70 mg/dL). These parameters integrate the dosage of HbA1c; however, that remains the gold standard for the evaluation of glycemic profile and the risk of long-term complications in patients with T1D but does not provide any information about the glycemic variability and daily patterns. According to the International Consensus of the ATTD Congress [48], there are standardized parameters derived from the report of CGM data using tools such as the Ambulatory Glucose Profile (AGP). Among these parameters, the mean sensor glucose, estimated HbA1c, glucose variability (standard deviation and coefficient of variation), number of days with sensor (≥14 days), percentage of CGM wear time (>70%) and percentage of time in range (TIR 70–180 mg/dL), time above range (TAR >180 mg/dL) and time below range (TBR <70 mg/dL) are used in clinical practice. Studies have shown that a TIR of 70% is correlated with an HbA1c of 7%. The International Consensus Guideline therefore recommends to reach these targets: TIR 70%, TBR <5% (<4%: <70 mg/dL, <1%: <54 mg/dL), TAR <30% (<25%: >180 mg/dL, <5%: >250 mg/dL) for patients with diabetes and in the general population. Many studies demonstrated the association between TIR and HbA1c, recommending an HbA1c goal of <7%. The use of standardized parameters derived from CGM data using tools such as AGP allows the identification of critical issues and to propose achievable therapeutic objectives. Therefore, the proposed targets for TIR, TBR, and TAR should be an essential component for CGM data analysis and treatment decisions [48].

## 4. Insulin Pumps

The first continuous subcutaneous insulin infusion (CSII) or insulin pump was introduced in 1979 [49]. Along with CGM, it had a significant technological development in recent years with the availability of integrated devices provided with algorithms that automatically suspend insulin infusion in case of current or predicted hypoglycemia (LGS or PLGS) to resume it when the hypoglycemia resolves or that adjust the basal insulin delivery rate based on glucose sensor data, leaving to the patient the administration of the bolus at mealtime, as in hybrid closed-loop systems. The insulin pump can be combined/integrated with a subcutaneous CGM device in real time. The association or integration of the two devices, called sensor-augmented pump (SAP) therapy, provides the glycemic profile in real time, with relative alarms, thus allowing the patient or caregiver to make immediate and retrospective adjustments to insulin dosing. SAP is more effective than multiple daily injections (MDI) with self-monitoring of blood glucose (SMBG) in improving glucose profile by reducing HbA1c without increasing the risk of hypoglycemia or severe hypoglycemia. CGM should be used at least 60% of the time to obtain better results. SAP systems with low glucose suspend (LGS) or predictive low glucose suspend (PLGS) function reduce the frequency and time spent in hypoglycemia as compared to integrated pumps, without leading to an increase in mean glucose levels, as demonstrated by HbA1c. For this reason, both LGS and PLGS are strongly recommended for all patients with T1D in order to reduce the severity and duration of hypoglycemia [50].

Compared to MDI, CSII reflects more faithfully the physiological insulin secretion and allows more precise and accurate dose adjustments. An Italian multicenter study analyzed the difference between MDI with or without a real-time sensor and insulin pump, demonstrating that glucose profile was better with continuous glucose monitoring (real-time CGM, rtCGM, Dexcom, San Diego, CA, USA) compared to discontinuous glucose monitoring (intermittently scanned CGM, isCGM, Flash Libre), and the results were even better with automated insulin delivery device [51]. Only 28% of patients with SAP, that is, an insulin pump not connected to CGM, reach the target of TIR > 70%. According to the ISPEDCARD registry 2021, only 30% of patients reach HbA1c < 7% (mean value 7.6 +/− 1.3%). According to the Austrian German Registry, the HbA1c value has slowly decreased overtime. The Sweet study (International study of benchmarking) shows that the trajectory of HbA1c has significantly decreased between 2008 and 2016 even if it does not reach 7%, which is the target value for the prevention of complications [52].

## 5. Hybrid Systems and Artificial Pancreas (AP)

Further development of the SAP are the hybrid systems (hybrid closed loop, HCL), in which the basal insulin infusion is modified in response to hyperglycemia or hypoglycemia prediction, and the fully automatic artificial pancreas (AP), in which there is an automatic connection between CGM and CSII through an algorithm which is installed on a chip or a device. The first experience of advanced technology for the management of children with T1D was born in Italy with the introduction of the artificial pancreas or advanced hybrid closed loop (AHCL) system, the Medtronic MiniMed 780G, that allows automated insulin delivery according to sensor glucose data provided by the CGM through a software integrated control algorithm. The technology of the AP employs an algorithm that bridges the CGM device with the insulin pump, thereby independently determining the dose of insulin needed without input from the user. Four algorithms are used in AP: proportional-integral-derivative (PID), model predictive control (MPC), fuzzy logic, and bihormonal algorithms. PID algorithms measure glucose levels and modify insulin infusion rate according to the difference between the measured glucose level and the glucose target point as expressed by proportional, integral, and derivative terms [53]. The Medtronic MiniMed 780 G is a model of AP paired with a Guardian 3 or Guardian 4 CGM that is designed with a PID algorithm with “insulin on board” and a license to use Fuzzy Logic. MPC algorithms predict future glucose values based on past trends and accordingly modify insulin infusion rates [54]. Taslim X2 Tandem with algorithm Control IQ is an AHCL system paired with a Dexcom G6 CGM device that, unlike Medtronic 780 G, uses an MPC algorithm. Fuzzy logic algorithms take advantage of the user’s or clinician’s therapeutic input through CGM [55]. Finally, the bi-hormonal algorithm control relies on both insulin and glucagon infusion [56]. Unlike HCL, AHCL has a major function that allows the administration of automatic correction bolus in case of hyperglycemia [57]. Several studies and a recent metanalysis highlight the advantage of this treatment option compared to standard SAP treatment in terms of glycemic control, time spent in hypoglycemia, and improvement in quality of life [58,59]. The two tables below summarize the main features and uses of the different technological devices for the management and treatment of diabetes (Table 1 and Table 2).

## 6. Impact of Technologies on Diabetes-Related Comorbidities

Diabetes care is focused on maintaining good metabolic control and reducing short- and long-term complications. The Diabetes Control and Complications Trial (DCCT) demonstrated that the lowering of hemoglobin A1c (HbA1c) levels by about 2% (9.0% to 7.1%) decreases the incidence of onset and progression of diabetic retinopathy, diabetic nephropathy and diabetic neuropathy by 47–54%, 39% and 60%, respectively, in both young adults (18–39 years old) [60] and adolescents (13–18 years old) [61] with a duration of diabetes of 1–15 years. The introduction of technologies and monitoring systems, which represent the standard of care for T1D, have led to a reduction in the morbidity and mortality associated with the microvascular and macrovascular complications of T1D, although these events have not been fully eliminated. When comparing complication rates approximately 20 years earlier with those of the DCCT/EDIC cohort after 20 years of follow-up, the cumulative incidences of proliferative diabetic retinopathy (PDR) and nephropathy decreased from 50% and 35%, respectively, to 30% and 12%, respectively; rates of end-stage renal disease (ESRD) requiring dialysis or transplantation have also decreased. Rates of other clinically serious complications have also dropped dramatically.

### 6.1. Cardiovascular Disease and Type 1 Diabetes

Cardiovascular disease (CVD) represents a more common cause of death than microvascular complications in patients with diabetes. In particular, subjects diagnosed before 10 years of age presented a 30-fold increased risk of coronary heart disease and acute myocardial infarction in early adulthood than healthy peers [62].

In T1D, hyperglycemia influences CVD through multiple mechanisms. In detail, hyperglycemia enhances in cells the formation of diacylglycerol (DAG), a key activator of protein kinase C (PKC). Augmented PKC activation determines the increased synthesis of matrix proteins (such as fibronectin and collagen), transforming growth factor (TGF)-β, that stimulate the thickening of the basement membrane; pro-inflammatory cytokines; vascular endothelial growth factor (VEGF), stimulating angiogenesis and vascular permeability; plasminogen activator inhibitor (PAI)-1, that prevents fibrinolysis; and reactive oxygen species (ROS) with consequent activation of oxidative stress that destroys arterial walls [63,64]. In addition, oxidative stress stimulates endothelial dysfunction by reducing the synthesis of NO, a crucial endothelial vasodilator [65].

Furthermore, hyperglycemia activates the polyol pathway, which converts excess intracellular glucose to sorbitol via the enzyme sorbitol dehydrogenase, finally resulting in the induction of intracellular oxidative stress with additional detrimental effects on arterial walls [66]. Moreover, chronic hyperglycemia activates non-enzymatic glycation of proteins, determining the formation of advanced glycation end-products (AGEs), interacting with the arterial wall through the related receptors (RAGEs) expressed on endothelial cells, thus exacerbating atherosclerosis. AGE/RAGE binding activates oxidative stress and NF-kB with the consequent trigger of inflammatory signaling, enhanced endothelium permeability, and endothelium dysfunction. NF-kB involvement determines by endothelial cells the expression of vascular cell adhesion molecule (VCAM)-1, intercellular adhesion molecule (ICAM)-1, and monocyte chemoattractant protein (MCP)-1, all contributing to increase the adhesion and attraction of monocytes and leucocytes [67,68,69]. Moreover, in endothelial cells, AGE/RAGE binding determines the production of endothelin-1, a strong vasoconstrictor [70]. AGEs also decrease the level of NO, a crucial endothelial vasodilator. AGEs trigger LDL oxidation as well as the formation of AGE-modified LDL (AGE-LDL) that, taken up by macrophages, leads to the secretion of proinflammatory cytokines, such as interleukin (IL)-1, IL-6, and TNF-α together with the of foam cell and atheromatous plaque formation. Interestingly, AGEs lead to thrombosis by enhancing the levels of tissue factor and decrease fibrinolysis by augmenting PAI-1 levels. Moreover, AGE/RAGE binding promotes smooth muscle cells proliferation and activation [71]. AGEs following the interaction with extracellular matrix proteins changes their turnover, with consequent extracellular matrix dysfunction and reduced flexibility of arteries [67,72]. In T1D patients, AGE pentosidine levels have been linked to coronary artery calcification [73]. Finally, in T1D patients, increased levels of methylglyoxal, the AGE main precursor, were linked to CVD in a 12-year follow-up study [74]. In addition, methylglyoxal has been linked to human carotid rupture-prone plaques [75]. It has also been demonstrated that hyperglycemia per se leads to endothelial dysfunction [76]. The main modifiable risk factor for decreasing CVD is represented by glycemic control. Current T1D treatment strategies and goals are based on the results of several studies of the DCCT and its epidemiological follow-up study, the Epidemiology of Diabetes Interventions and Complications (EDIC), which demonstrated that intensive insulin therapy aims to achieve glycemic control as close as possible to normoglycemia and is effective in delaying the onset and slowing the progression of microvascular and macrovascular complications observed in T1D [77]. There is evidence for a cardiovascular advantage of intensive glycemic control after long-term follow-up of cohorts affected with T1D. In the 9-year post-DCCT follow-up of the EDIC cohort, participants previously randomized to the intensive arm had a significant 57% reduction in the risk of myocardial infarction, stroke, or cardiovascular death compared with those previously randomized to the standard arm [78]. The benefit of intensive glycemic control in this cohort persisted for several decades and was associated with a reduction in all-cause mortality. Chronic hyperglycemia can promote atherosclerosis, endothelial dysfunction, and arterial stiffness [79]. Furthermore, an association between glycemic variability, CVD, and mortality, irrespective of mean glucose concentration, has been demonstrated [80]. T1D impairs endothelial function since childhood; in particular, the glucose variability can increase the vascular proliferation of smooth muscle cells, whereas hyperglycemia and the increase in fatty acid levels enhance oxidative stress and the production of advanced glycation end products [81,82]. In addition, hypoglycemia also contributes to cardiovascular complications. Hypoglycemia results in changes in hemodynamics, coagulation, arterial wall stiffness, cardiac electrophysiology, and autonomic function, explaining the observed associations between hypoglycemia and cardiovascular complications, including myocardial ischemia and cardiac arrhythmias [83]. Interestingly, subjects affected with T1D are more than twice as likely to experience cardiovascular mortality than the general population, even when they meet glycemic goals [84]. The progression of atherosclerosis begins in childhood, and young people with T1D can develop subclinical CVD even within the first 10 years of diabetes diagnosis [85]. CVD contributes to 25–50% of deaths in T1D subjects of less than 20-year diabetes duration, and this proportion increases with longer diabetes duration. DCCT/EDIC demonstrated that tighter glycemic control can improve cardiovascular risk factors, such as hypertension and carotid intima-media thickness, and even reduce cardiovascular events [60,62]. CGM devices and diabetes technologies, by improving glycemic trends and stability, may also have favorable impacts on T1D-associated complications. A large study from the Diabetes-Patienten-Verlaufsdokumentation (DPV) registry involving multiple diabetes centers in Germany, Austria, Switzerland, and Luxembourg found that the early initiation of insulin pump therapy within 6 months of diagnosis in people with childhood-onset T1D was associated with a better cardiovascular risk profile compared to those with delayed CSII initiation within 2–3 years of T1D diagnosis [86]. A reduction in the mean systolic blood pressure and an increase in high-density lipoprotein cholesterol (HDL-C), without significant relationships with diastolic blood pressure, low-density lipoprotein cholesterol (LDL-C), or triglycerides was observed [86]. Similar results were observed in a large T1D Swedish registry, which found that pump use was associated with a 45% reduction in coronary heart disease, 42% reduction in CVD, and a 27% reduction in all-cause mortality as compared to MDI use over a mean follow-up period of 6.8 years [87]. The decrease in cardiovascular mortality has been hypothesized to be related to a reduction in severe hypoglycemic episodes seen with insulin pump use. Less cardiovascular events, and lower mortality has been also associated with a longer duration of CSII use in T1D subjects [88]. A recent study compared indices of vascular function and myocardial performance in T1DM adolescents on MDI versus CSII. Infra-renal abdominal aorta (APAO), common carotid intima-media thickness (cIMT) and flow-mediated dilatation (FMD) represent the best non-invasive modalities to assess the vascular function and indirectly the myocardial status [89,90]. The mean cIMT and APAO values were higher in T1D patients than controls, and in those on MDI compared to those on CSII treatment [91]. Thus, the improving in glycemic control though CSII may reduce vascular alterations in T1D subjects.

### 6.2. Diabetic Nephropathy

Diabetic nephropathy (DN) indicates a specific kidney disease directly related to a long duration of diabetes and is often confirmed by histological lesions [92]. From 2002 to 2013, the prevalence of DN in children with T1D increased from 1.48 to 2.32 per 1000 [93], data only in part explained by a parallel increase in T1D prevalence [94]. Despite that, advanced DN is uncommon in pediatric patients, and the pediatrician’s challenge is to quickly detect early signs of renal involvement. The natural history of renal involvement in patients with T1D goes through several stages, from simple renal hypertrophy to several histological changes, which are the basis for an initial microalbuminuria (MA) followed by overt proteinuria that culminates in end-stage kidney disease [48]. The screening for detection of MA, as the earliest sign of DN, is recommended annually from puberty, at 10 years of age, and 5 years after T1D diagnosis [95]. Poor glycemic control, age at onset, duration of diabetes, puberty, high blood pressure, cigarette smoking, hyperlipidemia, genetic predisposition, and family history of diabetic complications are widely recognized as risk factors for developing DN [96]. In particular, puberty accelerates the development and progression of MA, conferring a three- to four-fold increase in the risk of MA after adjusting for other major risk factors [97].

DN development is primarily regulated by three pathways: (1) polyol and activation of PKC pathway. In detail, PKC pathway activation enhances the permeability of capillaries and triggers cellular stress and the expression of and transforming growth factor β1 (TGF-β1), exacerbating kidney injury [98]. Furthermore, polyol pathway activation alters the intracellular tension, enhances glycation as well as oxidative cell damage, and decreases anti-oxidation [99]. (2) Synthesis of AGEs in hyperglycemia breaks glomerular activity and leads to macrophage activation. In the kidney, AGEs/RAGE interaction determines chronic inflammation and oxidative stress, leading to kidney damage [100]. (3) Hyperglycemia leads to intraglomerular hypertension and glomerulus glomerular hyper filtration by triggering the local renin-angiotensin-aldosterone system. In the glomerulus, the elevated blood pressure increases renal vascular complications. Additionally, angiotensin II can lead to podocyte damage by augmenting ROS production [101]. Fibroblast Growth Factors (FGFs) also seem to have a key role, although further studies are needed. DN enhances the serum levels of the two most important members of this family of proteins, FGF21 and FGF23, which are positively related to renal damage. Consequently, FGF21 and FGF23 represent key biomarkers in order to predict renal disease progression, particularly in the early stage of DN [102].

Advances in technology for diabetes management have also led to improved pediatric nephrological outcomes via better glycemic control. The intensive treatment followed by the adolescent subgrouping in the DCCT study (in which the CSII) was associated with a reduction in the risk of developing MA by 10% compared to conventional treatment [61]. The protective effect of the intensive treatment on the development of nephropathy was maintained even during the 5–7 years of follow-up [103]. A higher TIR, a parameter associated with a lower risk of developing MA [104], can be easily obtained by the simultaneous use of real-time CGM and insulin pumps compared to intermittently scanned CGM and MDI [51]. However, whether CSII is preferable to MDI to ensure better glycemic control and decrease the risk of microvascular complications in the pediatric population is still a debated issue [105,106,107]. Schiel et al. showed that, in a cohort of 901 patients (age 11.5 ± 4.0), there were no differences between patients with CSII and MDI in respect of HbA1c, the mean amplitude of blood-glucose excursions, blood pressure, creatinine, and microalbuminuria [107]. These results were confirmed in a randomized control trial conducted by Blair et al. on 293 patients (median age of 9.8 years (range 0.7–16 years) that received CSII or MDI, without differences in terms of clinical benefits at 12 months of follow-up [106]. The artificial pancreas, a technology that minimizes user input by bridging continuous glucose monitoring and insulin pump treatment, is counted among the most innovative systems to manage diabetes. Karageorgiou et al. summarized in a meta-analysis the superiority of this system compared to the standard sensor-augmented pump in the treatment of T1D pediatric patients, but further studies on the impact on microvascular complications are needed [108].

### 6.3. Diabetic Retinopathy

Diabetic eye diseases are a group of eye problems that affect people with T1D, and they include diabetic retinopathy (DR), diabetic macular edema, cataracts, and glaucoma. DR involves the growth of abnormal blood vessels in the retina and is considered the most severe entity that carries the risk of blindness in T1D patients. Pathological glucose metabolism has primary and secondary consequences on the retina [109]. Primary consequences are derived from the altered glucose and lipid metabolism that directly influences retinal cells such as neural cells, glia, microglia, Müller cells, and vascular cells, together with pericytes, endothelial cells, and intravascular cells. T1D secondary consequences on the retina arise from the primary insults. DR arises from different mechanisms; in detail, pro-inflammatory changes happen in the retina that involve NO, leukotrienes, cyclo-oxygenase [110], VEGF together with hyperglycemia itself [111] as well as the low activated state of circulating leukocytes. In detail, hyperinsulinemia and hyperglycemia can enhance CD40 levels in platelets and monocytes [112], whereas CD40 expression inhibition downregulated both leukostasis and ICAM-1 expression in endothelial cells [113,114]. High levels of CD40/CD40L, Toll-like receptors, ER stress, CCR5, and the CD11b+CCR5hi monocyte are implicated in the early onset of leukostasis [115] in T1D murine models. Consistently, in diabetes, the pro-inflammatory monocyte phenotype, with enhanced CD80 levels, has been reported [116]. The Epidemiology of Diabetes Interventions and Complications and The Diabetes Control and Complications Trial showed that optimal T1D management significantly reduces the risk of development of DR, as demonstrated by the reduction of DR prevalence from 14–20% before the year 2000 to 3.7–6% after 2000 [61,117,118]. Despite the pediatric population being the one with the lowest risk of DR, the related literature refers to a prevalence ranging from 2.3% to 44% [119,120]. Whereas the risk of developing DR is minimal in children under 10 years old, puberty is considered the most important risk factor for developing and progressing retinopathy [121,122]. The duration of T1D after menarche was related to a 30% excess risk of developing DR compared with T1D duration before menarche [121]. In childhood, the risk of developing DR is also related to diabetes duration [123,124] and glycemic control, as demonstrated by a large study from the United States that showed an increase of 20% (95% CI 6–35%) of the DR risk for every 1-point increase in HbA1c in children with T1D [125]. The technological evolution in diabetes screening and treatment has also impacted the natural history of DR in young people. The standard DR screening method, consisting of the dilated eye exam, is giving way to the digital fundus photography, obtained by non-mydriatic fundus cameras connected via telemedicine to teleretinal networks. This innovative screening method can safely and quickly be performed by non-specialist-trained operators without the need for pupil dilation. It has been shown to increase screening rates, reduce the distance traveled for screening, and be more sensitive than classical mydriatic ophthalmoscopy [126,127]. Furthermore, the Food and Drug Administration approved 2018 the first autonomous artificial intelligence system for DR screening [128], which has been shown to have 85.7% sensitivity and 79.3% specificity in pediatrics, but at this time, it is approved for use only in adults [129]. Recent advances in technology, through a better glycemic control, have also led to improvement in the DR pediatrics outcomes. Wysocka-Mincewicz et al. studied 175 children (mean age 12.74 ± 3.7SD) with optical coherence tomography angiography and found a significantly lower fovea superficial vessel density, whole deep vessel density, parafovea deep vessel density and a larger foveal avascular (four early markers of DR) in the CSII vs. MDI group [130]. Additionally, Zabeen et al. found lower rates of retinopathy (OR 0.66, 95% CI 0.45–0.95, *p* = 0.029) in CSII group vs. MDI group of 989 patients (aged 12–20 years with a diabetes duration >5 years) [131]. Despite the verified role of continuous glucose monitor (CGM) in the improvement in glycemic control, few data are actually available on the effects of CGM use on development of DR in young people [132].

### 6.4. Diabetic Neuropathy

Diabetic peripheral neuropathy (DPN) is one of the main chronic microvascular complications of T1DM, which can lead to foot ulcers and lower-extremity amputations [133]. Generally, it occurs after at least 10 years of disease duration, and glycemic control represents the most important aspect of DPN management [134,135]. Clinical manifestations of DPN vary according to the type of nerve (large or small fibers) and organ involved (heart, bladder, intestine, etc.) [136]. DPN can develop as proximal asymmetric painful motor neuropathy, mononeuropathy, symmetric sensory-motor axonal neuropathy, and autonomic neuropathy [137]. However, symmetric sensory-motor axonal DPN shows the highest prevalence [137]. Axonal degeneration with demyelination has been reported in nerve biopsies [138,139].

In detail, damage to the Schwann cells and myelin sheath has been reported, with Schwann cells dissociating from axons both in unmyelinated and myelinated neurons [140]. Therefore, axonal impulse conduction and signaling are altered, and neurotrophic factors are reduced, leading to centripetal degeneration and distal axonal loss [22], with the longest nerve fibers at major risk of damage [140,141]. Different mechanisms have been proposed for DPN development, such as nerve barrier disruption and inflammation. The peripheral nerve microvessels are covered by a blood–nerve barrier (BNB). This barrier encloses the endothelial cells, pericytes, and basal lamina [142,143] and constitutes an important structure for transporting nutrients and protecting nerves [144]. The altered BNB function represents the first marker of damage associated with DNP development and progression. The increased permeability allows the transfer of high-molecular-weight proteins, such as immunoglobulin G and albumin, into the endoneurium [145,146]. The polyol pathway determining the hyperglycemia-induced flux alters membrane permeability and consequently molecule and electrolyte transport, perineurial basal or external laminae thickening, and thus edema. The last event determines subsequent ischemic nerve damage [147].

Cytokines, inflammatory cells, and growth factors are mediators of DNP development. In detail, hyperglycemia activates the cyclooxygenase-2 (COX-2) pathway in micro-vessels, leading to the development of oxidative and inflammatory stress in peripheral nerves [148]. A crucial role in DN development is determined by elevated AGE levels; consistently, AGEs are highly expressed in hyperglycemic status [149]. Additionally, an autoimmune etiology has been proposed for DNP but requires further investigation.

Emerging evidence suggests that glycaemic variability may be a crucial factor in the pathogenesis of DPN. The data from DCCT showed that intensive glucose monitoring decreased the incidence of DPN by 69% at five years [60]. A Cochrane systematic review and meta-analysis analyzed 17 randomized controlled trials (RCTs) examining the role of glycemic control in the prevention of DPN (seven T1DM subjects, eight in people with type 2 diabetes (T2DM), and two in both). Improved glycemic control significantly reduced the risk of DPN in T1DM but not in T2DM subjects [150]. This difference could be due to heterogeneity in DPN assessments across trials. At present, the data about the impact of diabetes technology on the pathogenesis of DPN are few; furthermore, most of the studies performed are cross-sectional and use different systems for the assessment of DPN [151]. Longitudinal studies are needed to establish the role of CGM in the delay of the onset of DPN.

### 6.5. Impact of T1D on Bone Health

T1D patients displayed a high risk of developing fractures with respect to the general population [152,153,154,155]. Newly diagnosed T1D can appear between the ages of 9 and 14 years [156], and childhood and adolescence represent crucial periods for optimal bone development [157], thus explaining the underlying abnormalities of bone health in these patients. In parallel, T1D is also associated with strong alterations in body composition, adiposity, and bone marrow adiposity [158,159,160,161]. Consistently, Abdalrahaman et al. reported that young women with childhood-onset T1D displayed a deficit in trabecular bone microarchitecture [162]. A detailed study has also been performed in T1D children and adolescents, with 10 out of 32 on CSII [90]. The authors showed that serum bone-specific alkaline phosphatase, C-terminal telopeptide of type I collagen (CTX), and total body (TB) and lumbar spine bone mineral density (BMD) SDS were lower compared with controls. Pediatric T1D patients also showed lower trabecular volume and trabecular numbers together with higher trabecular separation than controls. Although marrow adiposity was higher in patients than in the controls, even if not statistically significant, the marrow adiposity was inversely related to the trabecular number and directly to the trabecular space. Interestingly, they also demonstrated a positive correlation between the trabecular number and insulin dose, thus sustaining the role of insulin as an anabolic agent. In addition, the authors reported that bone formation was lower in children with poorer glycemic control but higher in children on CSII. Fractures appeared to a major extent in 31% of T1D children respect the 19% of controls. Moreover, the T1D children with a fracture history had poorer glycemic control and lower TB BMD with respect to T1D without fracture history [163]. Previously, we also demonstrated the key role of CSII with respect to MDI for both glycemic control and bone health [164,165]. In detail, we reported that glycemic control was better in CSII patients compared to MDI ones. Moreover, both glucose levels and HbA1c% were significantly decreased in CSII with respect to MDI patients. This improvement was also related to a major BMI-SDS and BMD in CSII with respect to MDI patients. The altered bone health in T1D is associated with the involvement of different biological effectors, such as Dickkopf-1 (DKK-1), sclerostin, and irisin.

#### 6.5.1. DKK-1 and Sclerostin

These represent two soluble inhibitors of the canonical Wnt signaling, a key pathway for bone-forming cell differentiation [166]. This signal involves the β-catenin translocation into the nucleus, where it can modulate the transcription of β-catenin dependent genes. In the absence of Wnt signal activation, β-catenin is degraded by the proteasome. This process allows to regulate the cytoplasmic concentration of β-catenin. To activate this signaling, the binding of the Wnt ligand to its Frizzled (FZD) receptor and Low-density lipoprotein receptor-related protein 5/6 (LRP5/6) co-receptors is necessary and determines the phosphorylation of LRP5/6 cytoplasmic tails and the recruitment of β-catenin destruction complex to the plasma membrane. The protein reorganization allows the formation of the signalosome, a multiprotein complex. It blocks β-catenin degradation; thus, β-catenin accumulates into the cytosol and translocates into the nucleus binding TCF/LEF family of DNA-bound transcription factors to dismiss transcriptional repression and activate the transcription of β-catenin responsive genes, and thus osteoblastogenesis [166]. DKK-1 binds with high affinity to either of the two binding sites on LRP6 or LRP5, thus preventing Wnt ligand binding and the formation of FZD-LRP5/6 complexes [167,168,169]. An additional mechanism for DKK-mediated inhibition of Wnt signaling includes the endocytosis of LRP6, which is determined by DKK concurrently interaction with LRP6 and the transmembrane receptors Kringle containing transmembrane protein 1 (KREMEN1) or KREMEN2. This interaction activates the quick LRP6 removal from the plasma membrane [170,171,172], thus further sustaining DKK-mediated inhibition of Wnt signaling. DKK1 has a crucial role, particularly during the initial stages of osteoblast commitment and differentiation [145], and then its expression decays. Consistently, DKK1 overexpression decreases Wnt signaling and thus blocks osteoblastogenesis and increases adipogenesis because these two cells share a common precursor that, according to the microenvironmental stimuli, will differentiate accordingly. Consequently, in vivo, DKK1 overexpression is associated with reduced bone formation and osteopenia [173,174]. Differently, reduced DKK1 expression determines bone formation enhancement with consequent high bone mass in young growing mice [174,175]. Similarly, reduced DKK1 binding affinity to LRP5 in certain LRP5 gain of function mutations resulted in a high bone mass phenotype in these patients [176].

Sclerostin (the product of the *SOST* gene) is a secreted glycoprotein that inhibits Wnt signaling following the binding to the LRP5/6 extracellular domain [145] and thus directly competes with ligand binding. Loss of *SOST* leads to augmented canonical WNT signaling activation with consequently enhanced bone formation, which was more evident in female mice [177,178]. Patients and mice with *SOST* homozygous loss-of-function mutations [179,180,181] manifest sclerostosis, severely augmented bone mass and density [182]. Differently, transgenic mice overexpressing *SOST* show reduced bone mass [183,184,185]. It is also known that mechanical stimulation decreases osteocyte *SOST* expression, thus stimulating osteoblastogenesis, whereas mechanical unloading enhances *SOST* levels, thus inhibiting Wnt signaling together with osteoblast differentiation and activity [186,187,188].

Sclerostin is mainly expressed by osteocytes deeply embedded inside the mineralized bone matrix [189]. This exclusive “location” leads to the development of a sclerostin-neutralizing antibody (Romosozumab) approved for the therapeutic treatment of osteoporotic patients at high risk of fracture [190]. Different authors demonstrated the altered levels of DKK-1 and sclerostin in T1D [191,192,193,194,195,196], but they did not evaluate the differences arising from the use of different devices for insulin administration. Whereas previously, we deepened this issue. In detail, we demonstrated higher levels of DKK1 and sclerostin, inhibitors of bone formation, in T1D patients compared with the controls, but interestingly, consistently with a better BMD simultaneously, DKK1 and sclerostin levels reached the controls’ level in CSII patients, whereas with respect to the control and CSII groups DKK1 and sclerostin levels were elevated in MDI group, further supporting the crucial role of the type of therapy on bone health and glycemic control in T1D patients [164].

#### 6.5.2. Irisin

Irisin originates from the Fibronectin type III domain, containing five proteins (FNDC5). This molecule in its structure has a signal peptide for ER [197], a hydrophobic transmembrane domain, a fibronectin III domain (that is the main part of irisin in the extracellular domain), and a carboxyterminal cytoplasmic domain. Following the N-glycosylation [198] in the ER and cleaving by disintegrin and metallopeptidase domain (ADAM) proteins (i.e., ADAM10) [199], irisin is secreted. This molecule is mainly secreted by skeletal muscle cells; initially, it was clarified its role in adipocyte trans-differentiation [200], and a few years later, its anabolic effect on bone (PNAS), leading to numerous related publications [201,202]. Myokine is involved in different bone diseases, including T1D [165], in which we explored irisin levels considering the different insulin devices. In detail, we reported the enhanced irisin levels in T1D patients compared to the controls, which correlated with both glycemic controls and bone status. In fact, irisin levels were negatively related to HbA1c%, years of diabetes, 25(OH)-Vitamin D, and positively with BMD and osteocalcin levels. Interestingly, in this case, we found the highest levels of irisin in CSII patients compared to MDI and control groups.

## 7. Estimated Life Expectancy

Recently, Gregory et al. published data about the global incidence, prevalence, and mortality of T1D in 2021 with projections to 2040. They reported that in 2021, there were about 8.4 million T1D subjects worldwide. In detail, 1.5 million (18%) were younger than 20 years, 5.4 million (about 64%) were 20–59 years old, and 1.6 million (19%) were 60 years or older. In that year, there were 0.5 million new cases diagnosed (median age of onset 29 years), and about 35,000 non-diagnosed subjects died within 12 months of symptomatic onset. One-fifth (1.8 million) of T1D subjects were in low-income and lower-middle-income countries. The remaining life expectancy of a T1D 10-year-old diagnosed in 2021 fluctuated from a mean of 13 years in low-income countries to 65 years in high-income countries. In 2021, missing prevalent cases were estimated at 3.7 million. In 2040, the authors predict an increase in prevalent cases to 13.5–17.4 million (60–107% higher than in 2021), with the major relative growth versus 2021 in low-income and lower-middle-income countries [203]. An additional study tries to estimate both the total and diagnosed T1D incidence worldwide and to project childhood indicators of T1D incidence from 1990 to 2050 for each country. They reported that in 2021, there were 355,900 total T1D new cases worldwide among children and adolescents, of which 56% (200,400 cases) were diagnosed. Estimated underdiagnosis significantly differs by region, with over 95% of new cases diagnosed in New Zealand and Australia, northern and western Europe, and North America, but less than 35% of new cases diagnosed in southeastern and south Asia, west Africa, and Melanesia. The total number of T1D incident childhood cases is estimated to augment to 476,700 in 2050 [204].

## 8. Conclusions

Diabetes technologies have greatly improved the management and care of people with T1D and the prevention of T1D-related complications. Glucose-sensitive automated insulin delivery systems, predictive insulin pump therapy for low glucose management, and hybrid closed-loop systems have resulted in improvements in glycemic control and reduced exposure to hypoglycemia. A milestone has been reached with algorithm-guided glucose-sensitive insulin delivery in the form of a hybrid artificial pancreas system. Over the next decade, advanced closed-loop systems with added data management capabilities will become the standard of care for people with T1DM across all age groups. However, bioartificial pancreas and “smart” insulin strategies will take much longer to demonstrate safety and efficacy in humans.

## Figures and Tables

**Table 1:** Technological devices for management and treatment of diabetes.

Advantages of CGM (rtCGM/FGM)	Use of rtCGM	Use of FGM	Advantages and Use of Insulin Pumps (CSII)	Advantages of AID Systems	Use of AID Systems (SAP, LGS, PLGS)
Accuracy and precision data Automatic setting of date, time Direct information on the diary tool, pre- and postprandial averages, standard deviation (SD) Alarms, reminders Trend indicators Prediction of HbA1c Bolus calculator Detection of ketonemia Data download Features of the specific software for a given instrument Pump connection Application for viewing data on smartphones connectivity	Recommended in the following conditions: - Asymptomatic, recurrent, severe hypoglycemia ≥2/year - Inadequate metabolic control and/or poor QoL - Need for simple and predictive alarms Others: > 10 SMBG/day agophobia	Recommended in the following conditions: - Lower risk of hypoglycemia - Inadequate metabolic control and/or poor QoL - Motivation to check the readings of the device several times a day Others: > 10 SMBG/day agophobia	Provide insulin delivery in two ways: - basal insulin delivery in a steady, measured, and continuous dose - surge (“bolus”) doses at mealtime Recommended in the following conditions: Inadequate metabolic control (elevated HbA1c, persistently above the desirable target and/or glycemic instability) despite intensive and optimized use of MDI High insulin sensitivity Risk of recurrent, nocturnal, or severe hypoglycemia Lifestyle impairment with MDI Newborn and preschool children Compliance of children, parents, and/or caregivers with technological devices	Increase in TIR, especially in the night hours, without an increased risk of hypoglycemia	Recommended in the following conditions: Frequent episodes of disabling and/or unrecognized severe hypoglycemia despite optimization of insulin pump therapy and SMBG Age < 6 years School age with frequent hypoglycemia, glycemic instability, or suboptimal glycemia Need for extremely frequent glycemic self-monitoring (>10/day) Compliance of children, parents, and/or caregivers with technological devices Not recommended if: No improvement in the risk of hypoglycemia and metabolic control, poor compliance, inability to wear the sensor continuously or to manage the “lag time”, interpret the trend arrows, perform the calibration correctly

AID: automate insulin delivery.

**Table 2:** Closed loop systems.

Pump Insulin Closed-Loop Term	Medtronic 670G Rapid Acting Auto Mode	Medtronic 780G Rapid Acting Smart Guard	Tandem t:slim X2 Ultra Rapid and Rapid Acting Control IQ
Algorithm	Treat-to-target proportional integral derivative with insulin feedback	Treat-to-target proportional integral derivative with insulin feedback; added fuzzy logic component	Treat-to-range predictive control
Set-up	TDD, weight, basal rates, ICR, ISF, active insulin time 7 days of manual mode	TDD, weight, basal rates, ICR, ISF, active insulin time 7 days of manual mode	TDD, weight, basal rates, ICR, ISF
Automated corrections used to supplement basal delivery	Based on total daily insulin dose last 2–6 days	Based on total daily insulin dose last 2–6 days	Based on pre-programmed basal rates
Automated insulin delivery	No	Yes, if glucose >120 mg/dL, and at maximum “auto basal” delivery	Yes (max 1/hour), if glucose predicted to be >180 mg/dL, delivers 60% of calculated dose
Glucose target	120 mg/dL Change to 150 mg/dL for set duration (30 min–12 h)	120 mg/dL Change to 150 mg/dL for set duration (30 min–24 h)	110–150 mg/dL Exercise range 140–160 mg/dL (manual start/stop only) Sleep range 110–120 mg/dL, prevents autocorrections
Adjustable setting	I:C ratio, active insulin time, glucose target	I:C ratio, active insulin time, “BG target” (algorithm target)	Basal rates, I:C ratio, ISF, glucose target range
No adjustable setting	Basal rates, ISF (automatically calculated and adapted)	Basal rates, ISF (automatically calculated and adapted)	Active insulin time (set at 5 h)
Exercise mode	No	Yes	Yes
Boost mode	No	No	No
Sick day rules	Recommended to revert to open loop for illness and/or ketone	Recommended to revert to open loop for illness and/or ketone	Recommended to revert to open loop for illness and/or ketone
Automatically reverts to open loop if…	Prolonged hyperglycemia, max/min insulin delivery, loss of CGM data, sensor integrity concerns, lack of calibrations.	Prolonged hyperglycemia, max/min insulin delivery, loss of CGM data, sensor integrity concerns, lack of calibrations.	Prolonged hyperglycemia (670 G only), max/min insulin delivery, loss of CGM data, sensor integrity concerns, lack of calibrations.
Meal bolus	Strenghten I:C ratios 10–25% Pre-meal boluses recommended for optimal outcomes	Bolus calculator automatically provides sensor glucose level for calculation or BG if performed in past 12 min Pre-meal boluses recommended	Bolus calculator automatically pro vides sensor glucose level for calcula tion If sensor glucose < 110, system will prompt to reduce carb bolus Pre-meal boluses recommended
Hypo treatment	Consider treating hypoglycemia with fewer carbohydrates (5 g CHO) depending on recent insulin delivery since insulin will likely have decreased or suspended	Consider treating hypoglycemia with fewer carbohydrates (5 g CHO) depending on recent insulin delivery since insulin will likely have decreased or suspended	Consider treating hypoglycemia with fewer carbohydrates (5 g CHO) depending on recent insulin delivery since insulin will likely have decreased or suspended
System optimization	Fingerstick BG check required. Enter into pump to give correction bolus If insulin sensitivity fluctuates widely overnight (e.g., young children), setting a temp target to prevent hypoglycemia No need to change/edit recommended bolus doses (algorithm has been likely giving more insulin in background) Extended/combo bolus function not available	Use of temp target will turn off automated corrections If insulin sensitivity fluctuates widely overnight (e.g., young children), setting a temp target to prevent hypoglycemia No need to change/edit recommended bolus doses (algorithm has been likely giving more insulin in background) Extended/combo bolus function not available	Use exercise activity, considering that auto-corrections will still occur Set sleep activity schedule overnight for tighter target Adjust doses for individuals with shorter active insulin time Extended bolus available, up to 2 h
Type of sensor	Guardian 3	Guardian 3	Dexcom G6
Calibrations	3–4 per day (before meals and at bedtime). Avoid when glucose fluctuates widely (e.g., after eating, after treating hypo, during exercise)	3–4 per day (before meals and at bedtime). Avoid when glucose fluctuates widely (e.g., after eating, after treating hypo, during exercise)	Rarely required (factory calibrated)
Sensor life	7 days	7 days	10 days
Remote monitoring	No	Yes, Carelink Connect app	Yes, Dexcom G6 Follow app (CGM data)
Upload/Data sharing	Manual downloading	Yes, MiniMed mobile app (pump and CGM data)	Yes, t:connect mobile app
Remote boluses	No, fingerstick BG check needed	Yes	Yes

TDD: total daily dose, ICR: insulin carbohydrate ratio, ISF: insulin sensitivity factor.
